# Certificates to Nostrums

**Published:** 1853-03

**Authors:** 


### CERTIFICATES TO NOSTRUMS.

Some years since, Dr. C. W. Crozier, of Knoxville, Tenn., submitted a plan to the profession for "superseding quack nostrums." An "Association of Physicians" had been formed, he said, which had undertaken the herculean labor. He wrote to the Senior Editor of this journal asking his opinion of the scheme, and submitting for his revision and criticism certain compounds. As Dr. Crozier's project seemed to be a benevolent one, a civil answer was returned to his letter, and out of this he has manufactured a certificate to the virtues of some pills which he is now advertising as a nostrum! If this is a part of his plan for "suppressing quackery," all we have to say is, that it appears to us as a queer one; and if such is the use he makes of letters procured by such a method, we assure him that gentlemen will be cautious hereafter how they put it into his power to use their names.

